# Effects of Grafting Degree on the Formation of Waterborne Polyurethane-Acrylate Film with Hard Core–Soft Shell Structure

**DOI:** 10.3390/polym15183765

**Published:** 2023-09-14

**Authors:** Yong Rok Kwon, Seok Kyu Moon, Hae Chan Kim, Jung Soo Kim, Miyeon Kwon, Dong Hyun Kim

**Affiliations:** 1 Materials & Component Convergence R&D Department, Korea Institute of Industrial Technology (KITECH), 143, Hanggaul-ro, Sangnok-gu, Ansan-si 15588, Republic of Korea; yongrok@kitech.re.kr (Y.R.K.); anstjrrb@kitech.re.kr (S.K.M.); coolskawk@kitech.re.kr (H.C.K.); kimjungsoo11@kitech.re.kr (J.S.K.); mykwon@kitech.re.kr (M.K.); 2 Department of Material Chemical Engineering, Hanyang University, 55, Hanggaul-ro, Sangnok-gu, Ansan-si 15588, Republic of Korea; 3 School of Integrative Engineering, Chung-Ang University, 84, Heukseok-ro, Dongjak-gu, Seoul 06974, Republic of Korea

**Keywords:** waterborne polyurethane-acrylate, 2-hydroxyethyl methacrylate, minimum film formation temperature, grafting, nano-indentation

## Abstract

Waterborne polyurethane-acrylate (WPUA) grafted with polyurethane was prepared to improve the film-forming ability of hard-type acrylic latex. To balance the film-formation ability and hardness, the WPUA latex was designed with a hard core (polyacrylate) and soft shell (polyurethane). The grafting ratio was controlled through varying the content of 2-hydroxyethyl methacrylate (HEMA) used to cap the ends of the polyurethane prepolymer. The morphologies of the latex particles, film surface, and fracture surface of the film were characterized through transmission electron microscopy, atomic force microscopy, and scanning electron microscopy, respectively. An increase in the grafting ratio resulted in the enhanced miscibility of polyurethane and polyacrylate but reduced adhesion between particles and increased minimum film formation temperature. In addition, grafting was essential to obtain transparent WPUA films. Excessive grafting induced defects such as micropores within the film, leading to the decreased hardness and adhesive strength of the film. The optimal HEMA content for the preparation of a WPUA coating with excellent film-forming ability and high hardness in ambient conditions was noted to be 50%. The final WPUA film was prepared without coalescence agents that generate volatile organic compounds.

## 1. Introduction

Acrylic latexes are widely used in coating formulations to reduce volatile organic compounds (VOCs) that impact the environment and human health [1]. However, solvent-based binders are still typically used in certain coating applications with high performance requirements, such as enhanced hardness, gloss, and scratch resistance [2]. These characteristics of waterborne coatings are inferior to those of solvent-based coatings due to their suboptimal film-forming ability. In waterborne systems, the latex polymer is dispersed in the form of nano- or micro-sized individual particles in the water phase. To achieve a continuous film during film formation, smooth coalescence must be ensured between these particles and chain diffusion [3]. Hard latexes can be used to achieve strong mechanical properties. However, their high transition glass temperature (*T_g_*) limits intergranular coalescence [4]. Owing to these aspects, it remains challenging to balance film formation and mechanical properties. Slow-drying solvents or low-molecular-weight plasticizers have been incorporated in waterborne coatings to facilitate polymer diffusion in the rigid latex [5,6]. However, such additives result in increased VOC emissions and adversely affect hardness development, chemical resistance, and durability [7].

Various methods based on *T_g_* heterogeneity, such as oligomer integration, the use of hard/soft latex blends, and the introduction of core–shell latexes, have been developed to simultaneously achieve low VOC emissions, high hardness, and low minimum film formation temperature (MFFT) [8,9,10]. The introduction of an oligomeric plasticizer that was prepared in situ using a chain transfer agent helped decrease the MFFT of the acrylic latex and VOC emissions by less than half [8]. In the case of hard/soft latex blends, the hard phase enhances the film hardness, and the soft phase promotes film formation [9]. Using a hard core–soft shell latex, a more uniform distribution than that of a blend can be achieved through incorporating the different phases into one structure. The improved mechanical and film-forming properties of acrylic core–shell latex depend on various parameters, such as the shell *T_g_*, shell specific gravity, and particle size [10].

An alternative approach is to introduce polyurethane (PU), known for its excellent flexibility and hydrogen bonding ability, into acrylic latex [11]. Wang et al. used a PU dispersion as a polymer coagulant to aid film formation and demonstrated its potential as an alternative to conventional coalescence aids [12]. However, the thermodynamic immiscibility between acrylic polymers and PU may lead to significant phase separation [13]. To overcome this problem, vinyl-terminated PU can be grafted onto acrylic chains. Grafted waterborne polyurethane-acrylate (WPUA) latexes have been noted to be superior to conventional blend latexes owing to improved compatibility [14]. However, excessive grafting can reduce the mobility of the polymer chains, thereby inhibiting interparticle coalescence.

In most of the existing studies, the effect of grafting on WPUA latex has been interpreted in terms of the phase separation and crosslinking density, and the film development process has been rarely considered [15,16,17,18,19]. In particular, no study has been reported on the design of WPUA latex with a hard core-soft shell structure to prepare hard coatings without coalescence additives that generate VOCs. Latex formulations that emit low VOCs and form films under ambient conditions are highly attractive to consumers. Therefore, this study was aimed at developing a latex coating formulation with excellent mechanical properties and film-forming ability in ambient conditions through introducing PU with high coalescing ability. To this end, we attempted to clarify the relationship between the grafting degree and film-forming ability of WPUA latex with a hard core–soft shell structure.

## 2. Experiment

### 2.1. Materials and Methods

Polytetrahydrofuran (PTHF, *M*_n_ = 1000 g/mol, Sigma Aldrich, St. Louis, MI, USA) and dimethylol propionic acid (DMPA, Sigma Aldrich, St. Louis, MI, USA) were dried in a vacuum oven at 60 °C for 24 h before use. Isophorone diisocyanate (IPDI), 2-hydroxyethyl methacrylate (HEMA), methyl methacrylate (MMA), and n-butyl acrylate (BA) were purchased from Sigma Aldrich, St. Louis, MI, USA. Dibutyltin dilaurate (DBTDL, Sigma Aldrich, St. Louis, MI, USA) was used as the catalyst for the urethane reaction. Triethylamine (TEA), ethylene diamine (EDA), ammonium persulfate (APS), and 1-methyl-2-pyrrolidinone (NMP) were purchased from Alfa Aesar, Ward Hill, MA, USA, and used as received.

### 2.2. Preparation of WPUA Samples

**Figure 1:**
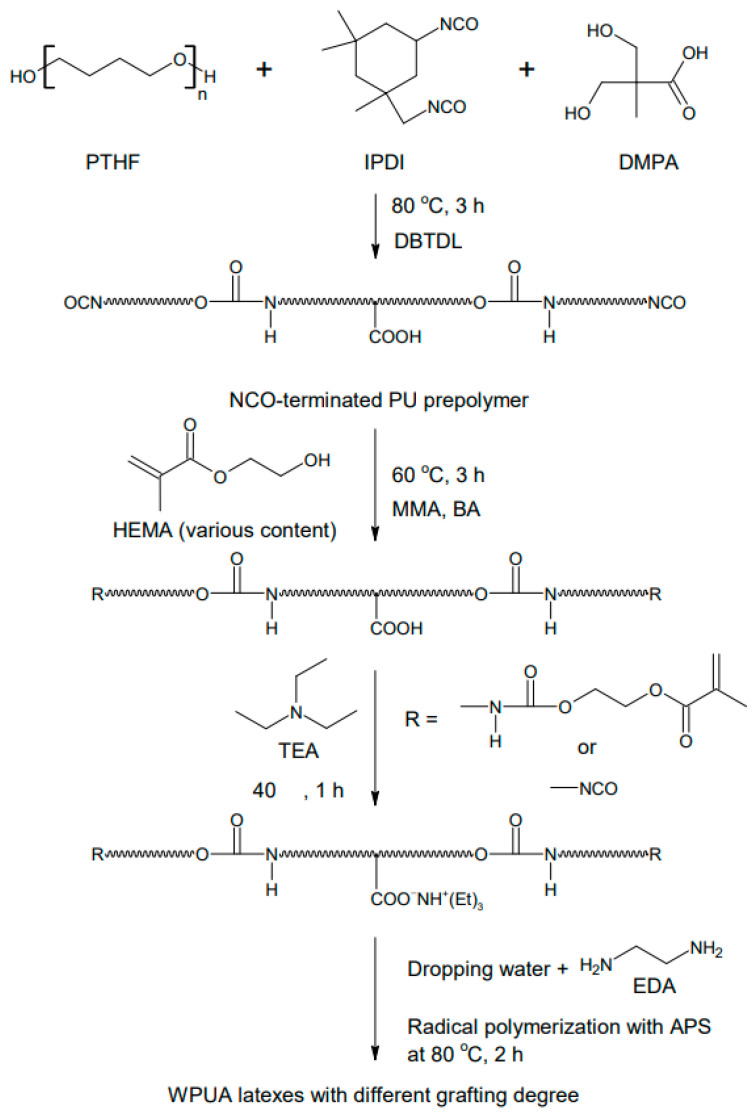
Synthesis process of WPUA latexes.

Figure 1 shows the synthesis process of WPUA latex. PTHF and IPDI were added to a 500 mL four-necked reactor and heated to 70 °C. The mixture was stirred with a mechanical stirrer under a nitrogen atmosphere. DMPA dissolved in NMP was added dropwise to the mixture and then stirred at 80 °C for 30 min. Subsequently, DBTDL was added, and the reaction was sustained until the NCO content reached the theoretical value. The resulting NCO-terminated PU prepolymer was cooled to 60 °C, and the viscosity was lowered through adding MMA and BA. Next, HEMA was added and allowed to react at the same temperature for 3 h to synthesize the acrylic terminal PU. The system was cooled to 40 °C, and TEA was added to neutralize the –COOH groups of DMPA for 1 h. The reactor was then immersed in an ice bath, and a mixture of distilled water and EDA was slowly introduced into the dropping funnel under high-speed agitation (600–700 rpm). After the completion of water dispersion and chain extension, the reactor was heated to 80 °C, and APS was added to polymerize the acrylic monomers. Finally, a WPUA dispersion with a solid content of 30 wt% was obtained.

The HEMA content was calculated using the theoretical molar number of the NCO groups in the PU prepolymer. The synthesized samples were labeled as WPUA_H_0.00_, WPUA_H_0.25_, WPUA_H_0.50_, WPUA_H_0.75_, and WPUA_H_1.00_, depending on the molar ratio of NCO_PU prepolymer_/OH_HEMA_ (Table 1).

### 2.3. Preparation of WPUA Films

The WPUA film was prepared through casting the dispersion onto a Teflon mold or glass plate and drying for 48 h at 25 °C and 24 h in a vacuum oven at 40 °C.

### 2.4. Characterization

#### 2.4.1. Particle Size Distribution and Zeta Potential

The particle size and zeta potential of the dispersions were measured using a particle size analyzer (SZ-100, HORIBA, Kyoto, Japan) after diluting the samples with distilled water. The measurements were conducted in triplicate at 25 °C, and the average values of the three trials were used as the final results.

#### 2.4.2. Transmission Electron Microscopy (TEM)

The morphology of the WPUA particles was observed using a Tecnai F20 G2 (FEI, Hillsboro, OR, USA) instrument operating at an accelerating voltage of 100 kV. The latex was diluted to a solids content of 0.1 wt% and dyed with 3 wt% phosphotungstic acid solution [20]. The mixture was adjusted to pH 7.5 using aqueous ammonia to stabilize the latex particles. A drop of the sample solution was deposited onto a copper mesh and allowed to dry at room temperature.

#### 2.4.3. Gel Fraction

To measure the gel content of the WPUA films, 1 cm × 1 cm samples were immersed in acetone for 48 h (initial weight, *W*_0_). Subsequently, the samples were dried in a 60 °C vacuum oven for 24 h to eliminate any residual acetone (dried weight, *W*). The gel content (*G*) was calculated as

*G* = *W*/*W*_0_ × 100 (%) (1)

#### 2.4.4. XPS

XPS measurements were performed using an X-ray photoelectron spectrometer (NEXSA, Thermo Fisher Scientific, Waltham, MA, USA) equipped with an Al Kα X-ray source (1486.6 eV) to determine the elemental composition of the latex surface. Latex samples for XPS were prepared in powder form via lyophilization for 72 h.

#### 2.4.5. Fourier Transform Infrared Spectroscopy (FT-IR)

FT-IR spectroscopy (Cary 630, Agilent Technologies, Santa Clara, CA, USA) was performed to analyze the chemical structure of the WPUA. The spectra were recorded in the range of 650–4000 cm^−1^ at a resolution of 4 cm^−1^ with a scan count of 64. All measurements were obtained at room temperature in the attenuated total reflectance mode.

#### 2.4.6. MFFT

The MFFT measurements were obtained in the temperature range of 0–90 °C, following the ASTM D2354 standard [21], using custom-made MFFT equipment.

#### 2.4.7. Dynamic Mechanical Analysis (DMA)

The dynamic mechanical properties of the prepared films were characterized through a DMA (242C, Netzsch, Selb, Germany). Each film was heated from −80 °C to 120 °C at a rate of 0.66 K min^−1^ under a N_2_ atmosphere, with a frequency of 1 Hz.

#### 2.4.8. Atomic Force Microscopy (AFM)

The surface morphology of the WPUA film was investigated through tapping-mode AFM (XE-100, Park systems, Suwon, Republic of Korea). Topographic and phase images were observed in the 2 µm^2^ range at a scan rate of 1 Hz using a silicon probe.

#### 2.4.9. Scanning Electron Microscopy (SEM)

SEM (SU8000, Hitachi, Tokyo, Japan) was performed to observe the fracture surface morphology of the film. Observations were made at an accelerating voltage of 10 kV. Before the test, the film was frozen in liquid nitrogen and then fractured.

#### 2.4.10. Nano-Indentation Test

To evaluate the surface hardness and elastic modulus of the WPUA film, a load–indentation depth curve was obtained using a nanoindenter (ZHN, Zwick Roell, Ulm, Germany). Tests for each sample were conducted 10 times, and the average value was obtained according to ASTM D1474 [22].

#### 2.4.11. Cross-Cut Test

The adhesion of the WPUA coating to the substrate was evaluated using a cross-cutter, and the measurements were recorded according to the ISO 2409 standard [23].

#### 2.4.12. Transparency Evaluation

Transmittance was recorded on a UV–vis spectrophotometer (Lambda 35, Perkin Elmer, Waltham, MA, USA) in the visible range. Transmittance values were averaged after five measurements.

## 3. Results

### 3.1. FT-IR Analysis

**Figure 2:**
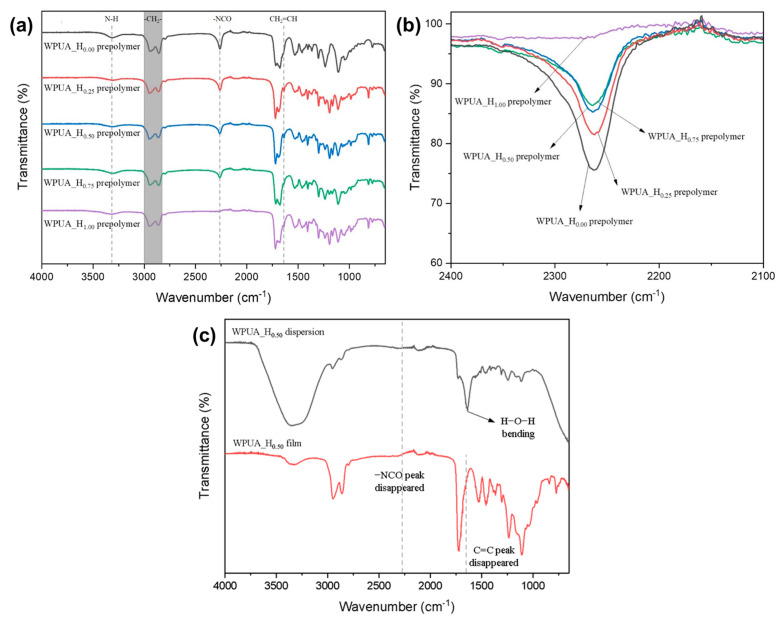
FT-IR spectra for the (**a**) overall frequency; (**b**) –NCO stretching region of the WPUA_H_0.00_, WPUA_H_0.25_, WPUA_H_0.50_, WPUA_H_0.75_, and WPUA_H_1.00_ prepolymers; and (**c**) WPUA_H_0.50_ dispersion and film.

Figure 2 shows the FT-IR spectra for the WPUA prepolymers, attained through the dibutylamine back titration method at the theoretical NCO content of the prepolymer. The peaks for all samples were similar, except for the peak corresponding to –NCO at 2270 cm^−1^ [20]. The disappearance of the peak associated with the –OH group at 3300 cm^−1^ confirmed the complete reaction of IPDI, PTHF, DMPA, and HEMA [24]. A broad peak pertaining to urethane N–H was observed at 3320 cm^−1^ [25]. The peak intensity associated with –NCO at 2270 cm^−1^ reduced with increasing HEMA content. Peaks corresponding to the symmetric and asymmetric stretching vibrations of –CH_3_ and –CH_2_ were observed at 2855–2955 cm^−1^ [26]. The peak associated with PTHF C–O–C stretching vibrations appeared at 1110 cm^−1^, whereas those for free C=O and hydrogen-bonded C=O appeared at approximately 1719 cm^−1^ [27]. The C=C peak for HEMA appeared at 1650 cm^−1^, indicating that no polymerization occurred during the 3 h reaction period [28]. At the end of all of the reactions, the residual –NCO and C=C peaks disappeared in the FT-IR spectra for the prepared WPUA_H0.50 dispersion and film (Figure 2c).

### 3.2. Particle Size and Gel Fraction of WPUA Latex

The average particle size, zeta potential, and gel fraction of the WPUA latex samples with different degrees of grafting are summarized in Table 2. Increasing the HEMA content resulted in an increase in the average particle size of the WPUA latex and decrease in the absolute zeta potential. During the synthesis of WPUA, a higher HEMA content facilitated the generation of additional grafting points between the acrylic polymer (PA, core) and PU polymer (shell). Consequently, the mobility of PU polymers with hydrophilic functional groups was reduced, which limited the localization of COO^–^ ions to the particle surface. This phenomenon reduced the number of ionic groups exposed on the WPUA latex surface, weakening the electrostatic repulsion between latex particles. As a result, agglomeration between WPUA latex occurred and particle size increased [29].

The gel fraction measurement results indicated that the acrylic terminal PU facilitated the crosslinking of the WPUA latex particles. As the HEMA content increased, the gel fraction of the latex rapidly increased. Theoretically, the PU chain is chain-extended with EDA to have a diacrylate structure, which can act as a crosslinking agent. The highest gel fraction (86.2%) corresponded to the WPUA_H_1.00_ sample, and highly crosslinked particles were formed.

### 3.3. Characterization of Core-Shell Structure

**Figure 3:**
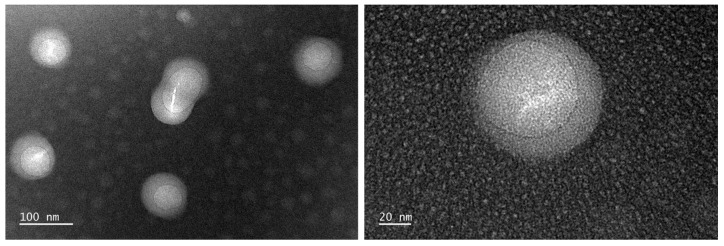
TEM images for the WPUA_H_0.50_ latex.

The particle morphology of WPUA_H_0.50_ latex was visualized in negative-stain TEM images as shown in Figure 3. The diameter of the particles was approximately 82 nm, which is similar to the results measured using the particle size analyzer. The latex particles exhibited a distinct core–shell structure, where the dark domains in the outer layer were PU and the relatively bright domains in the core were PA. The PU moiety is more polar than the PA moiety [30]. Therefore, shell regions with higher electron cloud density were observed to be darker.

**Figure 4:**
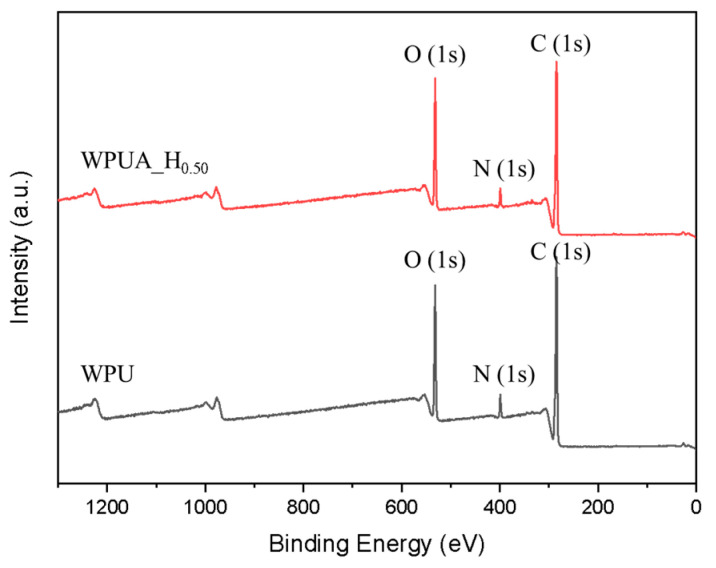
XPS spectra for WPUA_H_0.50_ and WPU particles.

The core–shell structure of WPUA was further characterized through comparing the surface elemental composition of WPUA and acryl-free WPU particles through XPS [31,32,33]. Here, WPU consists only of PU components in WPUA. WPUA_H_0.50_ and WPU latex were lyophilized prior to analysis to maintain the shape and morphology of the particles. Figure 4 shows the XPS spectra of WPUA_H_0.50_ and WPU particles. Although the WPUA_H_0.50_ sample contained approximately 58% acrylic component, it was almost identical to the surface component composition of the WPU particles. In addition, the contents of nitrogen elements associated with urethane and urea groups in WPUA_H_0.50_ (4.6%) and WPU (4.7%) were almost similar. These results proved that the hydrophobic acrylic component in WPUA is almost completely covered by the hydrophilic PU.

### 3.4. Viscoelastic and Thermal Characterization of WPUA Films

**Figure 5:**
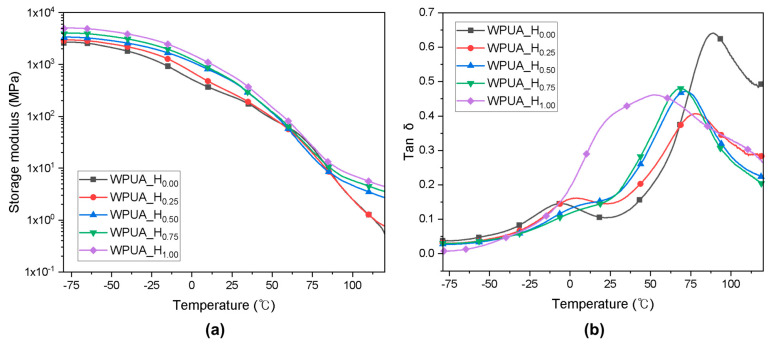
(**a**) Storage moduli and (**b**) tan *δ* vs. temperature curves for the WPUA_H_0.00_, WPUA_H_0.25_, WPUA_H_0.50_, WPUA_H_0.75_, and WPUA_H_1.00_ films.

Figure 5a shows the changes in the storage modulus of the WPUA films with increasing temperature. The storage modulus of the WPUA films increased as the HEMA content in the vitreous region increased, attributable to the higher degree of grafting and crosslinking. The storage modulus of WPUA_H_0.00_ at −70 °C was 2480 MPa, which increased to 5020 MPa at the same temperature. As the temperature increased, the molecular chain activity intensified, and the storage modulus gradually decreased. Notably, the storage moduli of the WPUA_H_0.00_ and WPUA_H_0.25_ rapidly decreased near 0 °C.

The crosslink density, the number of moles of elastically effective network chains per cubic centimeter of sample (*υ*_e_), can be determined through measuring the modulus in the rubbery region [34,35]. The network crosslink density of WPUA films with different HEMA contents was calculated from rubber elasticity theory using Equation (2) and is summarized in Table 3.

*υ*_e_ = *E*′/3*RT* (2)

where *υ*_e_, *E*′, *R*, and *T* are the crosslink density, elastic storage modulus, ideal gas constant, and temperature at which *E*′ is obtained, respectively. The elastic storage modulus of the film was obtained at 393.15 K (>*T_g_*). The calculated *υ*_e_ of WPUA films increased with increasing HEMA content.

Figure 5b shows the relationship between the loss factor (tan *δ*) and temperature, which can reflect the thermal behavior and degree of phase separation of the hybrid polymer film. All samples, except for WPUA_H_1.00_, exhibited two tan *δ* peaks, indicating the presence of two phases in the internal structure of the WPUA film [36]. The first phase transition, which occurred between −50 and 25 °C, was attributed to the chain activity of PU. The second phase transition, which occurred between 50 and 100 °C, was associated with the activity of the acrylic chain. The difference between the two peak temperatures decreased with increasing HEMA content, indicating improved miscibility between the PU and acrylic. WPUA_H_1.00_ exhibited the best phase miscibility, with a broad single peak curve between 0 and 90 °C.

Table 3 summarizes the *T_g_* and MFFT values of the WPUA samples obtained from DMA. The results indicate the clear dependence of the MFFT on the first *T_g_*. In general, a lower *T_g_* of the latex corresponds to superior interfacial adhesion and chain diffusion, resulting in a lower MFFT. The *T_g_* of the core acrylic polymer, calculated through the Fox equation for the prepared WPUA latexes, was 86 °C, which highlights that film formation is challenging in ambient conditions. In contrast, the MFFT values of WPUA_H_0.00_, WPUA_H_0.25_, and WPUA_H_0.50_ were lower than 25 °C, owing to the high adhesion capacity of the PU placed on the shell. PU is composed of soft segments, ionic groups, and urethane groups, and thus, it can be plasticized by water [37]. Consequently, the gaps between particles are filled, and chain entanglement is strengthened. Notably, the MFFT of WPUA_H_0.75_ and WPUA_H_1.00_ was significantly high, potentially because of the high degree of grafting and crosslinking. The covalent bonds between PU and acrylic polymer reduce the mobility of flexible PU chains, rendering it challenging to fill the voids between particles. Consequently, film cracking occurs, and a higher drying temperature is required to obtain a smooth film. Thus, although high-level grafting improves the compatibility of WPUA, it considerably limits its film-forming ability.

**Figure 6:**
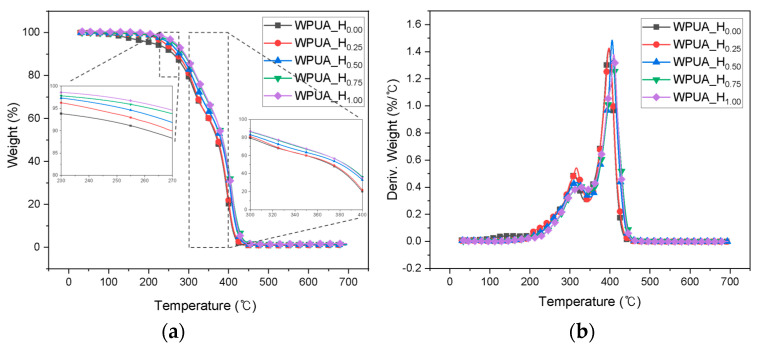
(**a**) TGA curves and (**b**) DTG graphs for the WPUA_H_0.00_, WPUA_H_0.25_, WPUA_H_0.50_, WPUA_H_0.75_, and WPUA_H_1.00_ films.

Figure 6 shows the TGA results for the WPUA samples. The initial weight loss values for the WPUA films with HEMA mol ratios of 0.25, 0.50, 0.75, and 1.00 were recorded at 240, 252, 262, and 264 °C, respectively, signifying the onset of the first decomposition temperature in the derivative thermogravimetry (DTG) graph. The temperatures corresponding to the first maximum degradation peaks were 310, 315, 315, 326, and 330 °C, associated with the decomposition of the hard segments of the PU [38]. The temperatures at which 50% weight loss was observed were 373, 381, 384, and 385 °C. The second maximum decomposition temperatures in the DTG analysis were 395, 396, 405, 408, and 409 °C, indicating the decomposition temperature of the soft segments and PA components [38]. Thus, as the HEMA content increased, the degradation temperature and maximum degradation temperature of the hard and soft segments increased by 20 ℃ and 14 ℃, respectively. This phenomenon was attributable to the grafting and crosslinking of WPUA, which resulted in improved heat resistance [39].

### 3.5. Morphological Characteristics of WPUA Films

**Figure 7:**
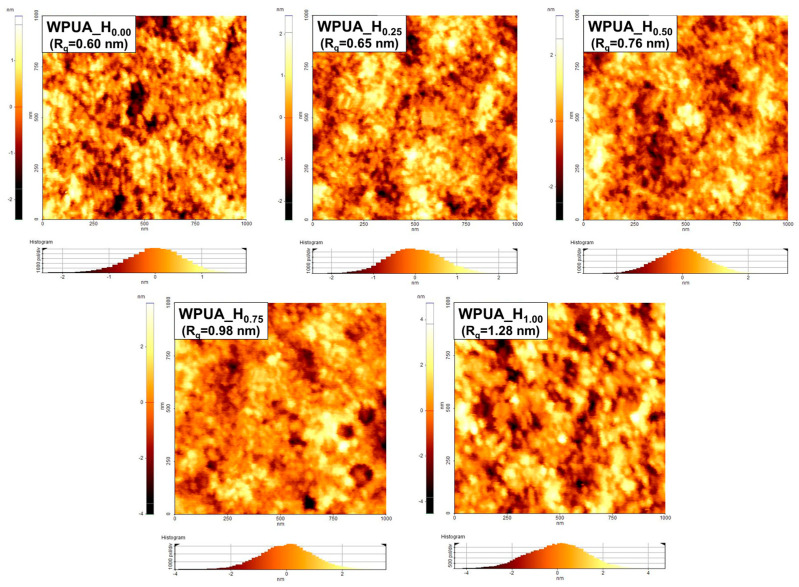
Tapping-mode-AFM height images for the WPUA_H_0.00_, WPUA_H_0.25_, WPUA_H_0.50_, WPUA_H_0.75_, and WPUA_H_1.00_ films.

To evaluate the coalescence and film-forming ability of the WPUA latex, the surface of the dried film was imaged via AFM. Figure 7 shows the height images and surface profile roughness values (*R*_q_) of the WPUA films with different HEMA contents. As predicted from the MFFT results, the increase in the HEMA content prevented the collapse of the particles and increased the surface roughness. The WPUA_H_0.25_ and WPUA_H_0.50_ samples, with a low degree of grafting, exhibited highly deformed surface morphologies. In contrast, most particles of the WPUA_H_0.75_ and WPUA_H_1.00_ samples, characterized by a high degree of grafting and crosslinking, did not collapse and maintained a spherical shape. Interestingly, smaller particles could be distinctly identified in the surface image of WPUA_H_0.00_. A potential reason for this phenomenon is that the shell polymer, free from the core, easily migrated to fill the voids between the particles, and undeformed core particles appeared on the surface [3]. Consequently, the WPUA_H_0.00_ film exhibited the lowest surface roughness.

**Figure 8:**
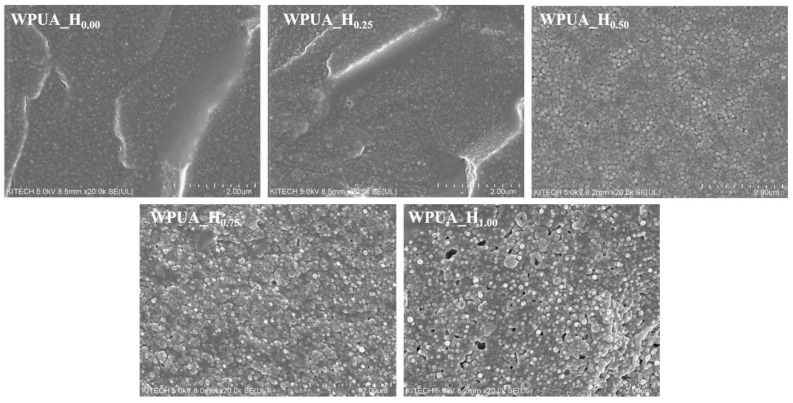
SEM images of the fracture surface for the WPUA_H_0.00_, WPUA_H_0.25_, WPUA_H_0.50_, WPUA_H_0.75_, and WPUA_H_1.00_ films.

Figure 8 shows the fracture surface SEM images of the WPUA samples. The results were consistent with those derived from AFM phase images. As the grafting ratio of WPUA increased, distinct latex particles and rough fracture surfaces were observed. Specifically, the WPUA_H_1.00_ film with the highest MFFT exhibited numerous micro-voids inside, attributable to its low particle-to-particle coalescence ability. Such discontinuous films may be characterized by several limitations, such as low barrier properties and mechanical strength.

### 3.6. Mechanical and Optical Properties

**Figure 9:**
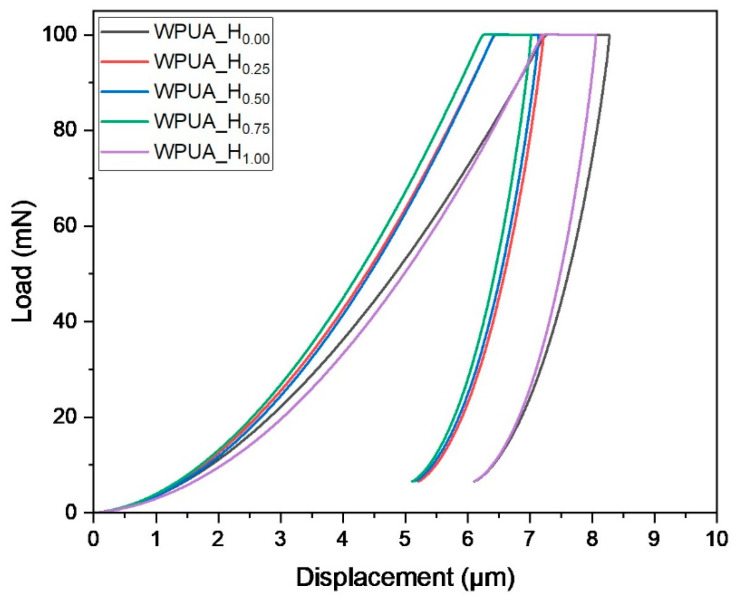
Typical load–indentation depth curves from nano-indentation tests on WPUA films.

The effect of the grafting ratio on the mechanical properties of WPUA coatings was investigated through nano-indentation and cross-cut tests. Figure 9 shows the load–indentation depth curve reflecting the mechanical surface behavior of WPUA coatings with different grafting ratios. Table 4 lists the average values of the indentation hardness (*H*_IT_) and modulus of elasticity (*E*_IT_). As the grafting ratio increased from 0 to 0.75, the indentation depth decreased at the same pressure. *H*_IT_ gradually increased from 67.50 MPa to 89.53 MPa, and *E*_IT_ increased from 2.87 GPa to 3.84 GPa. These trends were attributable to the strengthening effect induced by the high degree of grafting and crosslinking. Notably, the *H*_IT_ and *E*_IT_ of WPUA_H_1.00_ were 67.5 MPa and 2.87 GPa, respectively. The voids in the WPUA_H_1.00_ coating, as observed in the SEM images, resulted in decreased hardness and elastic modulus [40].

According to the cross-cut test results, the adhesion of the WPUA coatings decreased as the grafting ratio increased (Table 4), likely because of the film-forming ability of WPUA. In general, a greater deformability of the particles is associated with superior adhesion with the substrate. In the case of the WPUA latex particles with a low degree of grafting, the soft-shell PU polymer is expected to move freely and completely cover the substrate. These characteristics are associated with enhanced film-forming ability and superior adhesion of the WPUA coating.

In general, the transparency of composite polymers is closely related to the particle size, crystallinity of the polymer matrix, phase separation, and refractive index differences [41,42]. The grafted and crosslinked WPUA films exhibited excellent light transmittance in the visible range (Table 4). However, the WPUA_H_0.00_ film exhibited haze and recorded a 74% lower light transmittance. This is consistent with Desai’s report that the transparency of PU/poly(MMA) latex films without HEMA was reduced [43]. This is due to the difference in the refractive index between the highly separated PU and acrylic domains, as in the results obtained with DMA [14]. In particular, WPUA with a hard core-soft shell structure may have intensified phase separation due to the heterogeneity of *T_g_*. On the other hand, comparing WPUA_H_0.25_ and WPUA_H_0.75_, light transmittance increased even though the particle size increased from 70.5 to 84.5 nm. This may be because the improved compatibility had a greater impact on the transparency of the WPUA film than the particle size change in the 14 nm range. Therefore, improving the compatibility of the two phases is essential to obtain a transparent film. The reduced transparency of WPUA_H_1.00_ is attributed to the formation of voids.

## 4. Conclusions

This study was aimed at preparing coatings with excellent film-forming ability and high hardness at room temperature without the use of coalescence agents. To this end, PU was introduced into acrylic latex as a coalescence aid, and the effect of the grafting ratio of the core (PA) and shell (PU) on the properties and film-forming ability of WPUA was systematically investigated. Increasing the grafting ratio resulted in the crosslinking of the WPUA latex particles and an increase in their size. The grafted WPUA coating exhibited improved hardness, modulus, thermal stability, and compatibility. However, excessive grafting reduced the chain mobility of the soft shell, which limited the collapse and coalescence of the latex particles during film formation. Therefore, the MFFT of WPUA increased, resulting in the formation of a discontinuous film with reduced hardness and adhesion. These results highlight that the grafting ratio must be optimized to ensure a balance between the film-forming ability and mechanical properties of WPUA latex. In follow-up studies, we plan to prepare various series of PUs and investigate their coalescence-supporting abilities in more detail.

## Figures and Tables

**Table 1:** WPUA samples with different HEMA contents.

Sample	HEMA (mmol)	EDA (mmol)	OH_HEMA_/NCO_PUpre_ (mol Ratio)
WPUA_H_0.00_	0	30	0
WPUA_H_0.25_	15	22.5	0.25
WPUA_H_0.50_	30	15	0.50
WPUA_H_0.75_	45	7.5	0.75
WPUA_H_1.00_	60	0	1.00

PU prepolymer composition: PTHF = 0.02 mol, IPDI = 0.09 mol, DMPA = 0.04 mol, TEA = 0.04 mol; Acrylic composition: MMA = 61.2 g, BA = 6.8 g.

**Table 2:** Gel fraction, particle size, and zeta potential for WPUA latex.

Sample	Gel Fraction (%)	Particle Size (nm)	Zeta Potential (mV)
WPUA_H_0.00_	0	64.2	−72.4
WPUA_H_0.25_	5.8	70.5	−52.3
WPUA_H_0.50_	27.7	77.1	−42.9
WPUA_H_0.75_	53.1	84.5	−38.4
WPUA_H_1.00_	86.2	92.9	−35.2

**Table 3:** *T_g_*, *υ*_e_, and MFFT for WPUA films.

Sample	*T_g_*, PU (°C)	*T_g_*, PA (°C)	*υ*_e_ × 10^3^ (mol/cm^3^)	MFFT (°C)
WPUA_H_0.00_	−6.6	88.7	0.16	8.4
WPUA_H_0.25_	2.9	77.2	0.23	11.6
WPUA_H_0.50_	7.7	71.5	0.83	19.2
WPUA_H_0.75_	8.0	68.5	1.09	29.7
WPUA_H_1.00_	53.4		1.35	42.2

**Table 4:** Hardness, elastic modulus, and light transmittance of WPUA films.

Sample	*H*_IT_^a^ (MPa)	*E*_IT_^a^ (GPa)	Adhesion ^b^	Transmittance ^c^ (%)
WPUA_H_0.00_	63.0 ± 2.2	3.1 ± 0.1	5B	74 ± 2
WPUA_H_0.25_	84.9 ± 2.4	3.5 ± 0.1	5B	88 ± 2
WPUA_H_0.50_	86.4 ±3.2	3.5 ± 0.2	4B	90 ± 3
WPUA_H_0.75_	89.5 ± 4.7	3.8 ± 0.3	3B	91 ± 3
WPUA_H_1.00_	67.5 ± 5.1	2.9 ± 0.4	2B	87 ± 5

^a^ Results obtained from nano-indentation test; ^b^ results obtained from cross-cut test; ^c^ light transmittance in the visible range.

## Data Availability

The data are available upon request.
